# Identification and characterization of plasma proteins associated with intra-amniotic inflammation and/or infection in women with preterm labor

**DOI:** 10.1038/s41598-024-65616-x

**Published:** 2024-06-25

**Authors:** Hee Young Cho, Ji Eun Lee, Kyo Hoon Park, Bo Young Choi, Min Jung Lee, Da Eun Jeong, Sue Shin

**Affiliations:** 1 Department of Obstetrics and Gynecology, Seoul National University College of Medicine, Seoul National University Hospital, Seoul, Korea; 2 Chemical & Biological Integrative Research Center, Biomedical Research Division, Korea Institute of Science and Technology, Seoul, 02792 Korea; 3 Department of Obstetrics and Gynecology, Seoul National University College of Medicine, Seoul National University Bundang Hospital, 82, Gumi-Ro 173 Beon-Gil, Bundang-Gu, Seongnam, 463-707 Korea; 4 Department of Laboratory Medicine, Seoul National University College of Medicine, Seoul National University Boramae Hospital, Seoul, Korea

**Keywords:** Immunology, Biomarkers, Medical research

## Abstract

This study aimed to identify plasma proteins that could serve as potential biomarkers for microbial invasion of the amniotic cavity (MIAC) or intra-amniotic inflammation (IAI) in women with preterm labor (PTL). A retrospective cohort comprised singleton pregnant women with PTL (24–34 weeks) who underwent amniocentesis. Pooled plasma samples were analyzed by label-free liquid chromatography-tandem mass spectrometry for proteome profiling in a nested case–control study (concomitant MIAC/IAI cases *vs.* non-MIAC/IAI controls [*n* = 10 per group]). Eight target proteins associated with MIAC/IAI were further verified by immunoassays in a large cohort (*n* = 230). Shotgun proteomic analysis revealed 133 differentially expressed proteins (fold change > 1.5, *P* < 0.05) in the plasma of MIAC/IAI cases. Further quantification confirmed that the levels of AFP were higher and those of kallistatin and TGFBI were lower in the plasma of women with MIAC and that the levels of kallistatin and TGFBI were lower in the plasma of women with IAI than in those without these conditions. The area under the curves of plasma AFP, kallistatin, and TGFBI ranged within 0.67–0.81 with respect to each endpoint. In summary, plasma AFP, kallistatin, and TGFBI may represent valuable non-invasive biomarkers for predicting MIAC or IAI in women with PTL.

## Introduction

Preterm birth (PTB) occurs in 10–12% of all deliveries worldwide and is the leading cause of long-term neonatal morbidity and mortality; in particular, preterm labor (PTL) is responsible for one-third of all preterm births^1,2^. Although PTL is considered a syndrome with multiple causes, subclinical microbial invasion of the amniotic cavity (MIAC) and/or intra-amniotic inflammation (IAI) have been implicated as its major causes, with an overall incidence of 20–50% (10–20% for MIAC and 30–50% for IAI)^2–6^. Of note, MIAC and/or IAI (MIAC/IAI) directly increase the risk of early spontaneous PTB (SPTB) or adverse short- and long-term neonatal outcomes, along with indirect adverse effects on neonates by inducing preterm birth^6–13^. Thus, these conditions are likely to cause the worst outcomes in PTL. However, MIAC/IAI diagnosis requires invasive amniocentesis, a procedure that patients and clinicians may decline. Importantly, recent studies have shown that MIAC/IAI, particularly “mild sterile” IAI, can be effectively managed in a subset of patients with PTL using antibiotic therapy (in particular using clarithromycin)^14,15^. Thus, establishing non-invasive tools for the early and accurate diagnosis of MIAC/IAI is critical to ensure timely and effective therapy. Inflammatory cascade and its related signaling pathways are involved in the pathogenesis of PTL and subsequent SPTB^16,17^. Importantly, the inflammatory milieu of the amniotic cavity may be reflected by proteins in circulation^18–21^. Hence, plasma samples may represent a valuable tool for detecting MIAC/IAI biomarker using minimally invasive methods. Previous studies evaluated several potential inflammatory biomarkers in maternal blood collected from women with PTL and showed that several proteins (C-reactive protein [CRP], interleukin [IL]-6, adiponectin, and visfatin) are significantly overexpressed in the serum/plasma of women with MIAC/IAI complicated by PTL^22–25^. However, none of these inflammatory biomarkers, alone or in combination, are sufficiently sensitive or specific to predict MIAC/IAI in clinical practice.

Recent advances in proteomics technologies allow the assessment of a large portion of proteins/peptides present in the blood in an unbiased manner. Global plasma proteomic analysis can be performed to obtain information regarding the dynamic changes of the total proteome occurring during disease or infection, regardless of the its burden^26^. Previous studies on women with PTL attempted to use plasma proteomics to identify patients at risk of SPTB^27–29^. Moreover, in the context of preterm premature rupture of the membranes (PPROM), proteomic analysis is useful for identifying proteins associated with MIAC/IAI^30^. However, to the best of our knowledge, this approach has not been reported for the detection of MIAC/IAI women with PTL. The present study aimed to (i) identify potential plasma biomarkers related to MIAC and/or IAI in patients with PTL and intact membranes using large-scale quantitative discovery proteomics, (ii) characterize significant protein signaling pathway involved in the pathogenesis of these conditions, and (iii) determine whether the identified deregulated plasma proteins could be associated with SPTB occurrence within 7 days.

## Results

### Baseline characteristics of the discovery cohort

Table S1 shows the demographic and clinical characteristics of the discovery cohort used in the proteomic experiments. Because of matching, the participants of the case (both MIAC and IAI) and control (non-MIAC/IAI) groups were similar in terms of age, parity, gestational age (GA) at sampling, and medication use. The only exception was in the rate of antibiotic use, which was significantly lower in women with concomitant MIAC and IAI. The microorganisms isolated from the amniotic fluid (AF) samples of 10 patients with concomitant MIAC/IAI (case group) were *U. urealyticum* (*n* = 10) and *M. hominis* (*n* = 9), among whom 90.0% (9/10) of the patients with MIAC had polymicrobial infections.

### Discovery proteomic analysis

**Figure 1:**
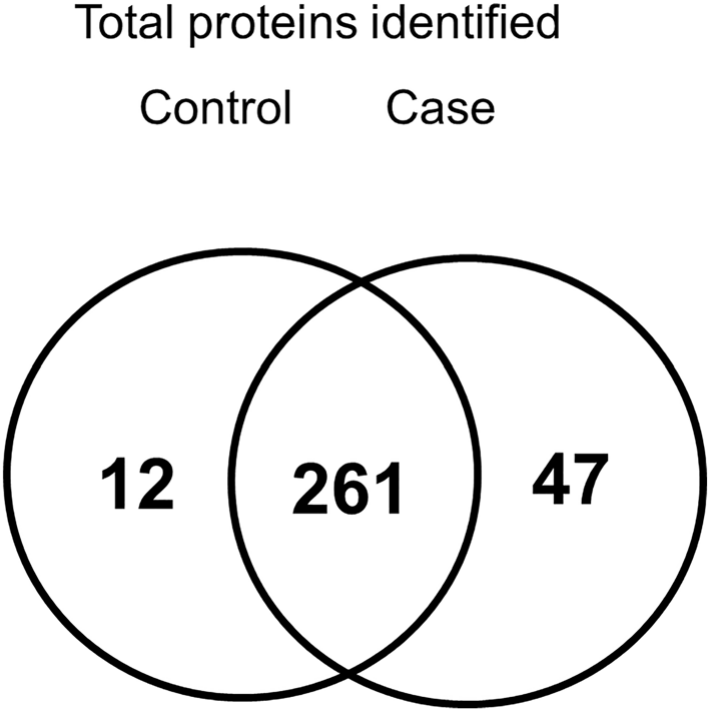
Venn diagrams with the number of identified proteins based on LC–MS/MS analysis. *LC* Liquid chromatography, *MS/MS* Tandem mass spectrometry.

Proteomics analysis revealed 273 and 308 proteins that were only present in the plasma of the control and case groups, respectively, whereas 261 proteins were present in both groups (Fig. 1). Thereafter, the following criteria were used to select differentially expressed proteins (DEPs) between concomitant MIAC/IAI case and non-MIAC/IAI control groups: (i) *P* < 0.05 determined by power law global error model (PLGEM); (ii) fold changes (FCs) ratio > 1.5 or < 0.66; and (iii) spectral counts > 50 from at least two proteomic technical replicates. Of 133 DEPs identified, 96 (72.2%) were upregulated and 37 (27.8%) were downregulated in the MIAC/IAI case group as compared with the non-MIAC/IAI control group (Table S2 and Fig. S1). Further analysis using ingenuity pathway analysis (IPA) revealed the top five canonical pathways that were mostly impacted during infection/inflammation in the amniotic cavity (Table S3): ‘acute phase response (APR) signaling’, ‘liver-X-receptor (LXR)/retinoid X receptor (RXR) activation’, ‘farnesoid X receptor (FXR)/RXR activation’, ‘IL-12 signaling and production in macrophages’, and ‘primary immunodeficiency signaling’.

### Baseline and clinical characteristics of the verification cohort (total study cohort)

In the total cohort (*n* = 230), the prevalence of MIAC and IAI was 8.6% (20/230) and 23.4% (54/230), respectively, and concomitant MIAC and IAI were observed in 8.2% (19/230) of the women. MIAC and IAI alone were present in 0.4% (1/230) and 15.2% (35/230) of the women, respectively, whereas 76.1% (175/230) of them exhibited neither MIAC nor IAI. The microorganisms isolated from the AFs of 20 PTL women with MIAC were genital mycoplasmas (*U. urealyticum* [*n* = 18] and *M. hominis* [*n* = 14]), *Streptococcus agalactiae* (*n* = 1), and gram-positive cocci (*n* = 1), among whom 70.0% (14/20) of patients with MIAC had polymicrobial infection.

Women with MIAC and IAI had significantly lower GA at sampling and delivery, higher serum CRP levels, higher rates of antibiotic use and histologic chorioamnionitis (HCA), and significantly or borderline significantly higher corticosteroid administration rates than those without these conditions (*P* = 0.051 and 0.006, respectively) (Table 1). The only exception was GA at sampling, which did not differ between patients with and without IAI but was significantly lower in those with MIAC. The rate of nulliparity did not differ between patients with and without MIAC, but tended to be lower in women with IAI than in those without this condition (*P* = 0.088) (Table 1).

**Table 1:** Demographic and clinical characteristics of the study population according to the presence or absence of microbial invasion of the amniotic cavity (MIAC) and intra-amniotic inflammation (IAI) in women with in women with preterm labor.

	MIAC		*P*-value	IAI		*P*-value
	Positive (n = 20)	Negative (n = 210)		Positive (n = 54)	Negative (n = 176)	
Maternal age (years)	31.8 ± 4.1	31.6 ± 4.1	0.949	32.1 ± 4.0	31.5 ± 4.1	0.293
Nulliparity	60.0% (12/20)	63.8% (134/210)	0.735	53.7% (29/54)	66.5% (117/176)	0.088
Gestational age at sampling (weeks)	28.2 ± 2.7	30.3 ± 2.5	**0.001**	29.7 ± 2.7	30.3 ± 2.5	0.126
Gestational age at delivery (weeks)	29.7 ± 3.8	35.4 ± 4.6	** < 0.001**	31.0 ± 3.4	36.1 ± 4.5	** < 0.001**
Serum CRP (mg/dL)	2.7 ± 2.0	0.7 ± 1.2	** < 0.001**	1.8 ± 1.8	0.6 ± 1.1	** < 0.001**
Use of tocolytic agents	100.0% (20/20)	91.9% (193/210)	0.374	92.6% (50/54)	92.6% (163/176)	1.000
Use of antibiotics	75.0% (15/20)	25.7% (54/210)	** < 0.001**	51.9% (28/54)	23.3% (41/176)	** < 0.001**
Use of antenatal corticosteroids	95.0% (19/20)	75.2% (158/210)	0.051	90.7% (49/54)	72.7% (128/176)	**0.006**
Clinical chorioamnionitis	5.0% (1/20)	0% (0/210)	0.087	1.9% (1/54)	0.0% (0/176)	0.235
Histological chorioamnionitis^a^	84.2% (16/19)	29.6% (32/108)	** < 0.001**	60.8% (31/51)	22.4% (17/76)	** < 0.001**

CRP, C-reactive protein. Data are given as mean ± standard deviation or % (n/N). ^a^Data for the histologic evaluation of the placenta were only available in 127 of the 230 women because in 22 cases, delivery took place at another institution and in 81 cases, histologic evaluation of the placenta was not performed because of our institutional policy that only the placentas in cases of preterm delivery are to be sent for histopathologic examination (*n* = 79) or because of missing data for the histological chorioamnionitis (*n* = 2).

Significant values are in bold.

### Verification of proteomic data in relation to MIAC in the total cohort

To verify the proteomic data, the expression pattern of eight DEPs (namely alpha-fetoprotein [AFP], galectin-3 binding protein [Gal-3BP], heat shock protein 70 [HSP70], kallistatin, lipocalin-2, pregnancy-specific beta-1 glycoprotein 1 [PSG1], S100 calcium-binding protein A8 [S100A8], and transforming growth factor beta-induced [TGFBI]) in the plasma was measured in 230 individual samples by enzyme-linked immunosorbent assays (ELISA). Notably, the median plasma levels of AFP were significantly higher and those of kallistatin were significantly lower in patients with MIAC than in women without this condition (Table 2). Moreover, the plasma TGFBI levels tended to be lower in women with MIAC (*P* = 0.084). These significant or borderline significant associations remained statistically significant in multivariate logistic models, even when adjusted for GA at sampling (Table 3). However, based on univariate analyses, no significant differences in plasma Gal-3BP, HSP70, lipocalin-2, PSG1, or S100A8 levels were observed in relation to MIAC in women with PTL (Table 2).

**Table 2:** Various plasma proteins of the study population according to the presence or absence of microbial invasion of the amniotic cavity (MIAC) and intra-amniotic inflammation (IAI) in women with preterm labor.

	MIAC		*P-*value	IAI		*P*-value
	Positive (n = 20)	Negative (n = 210)		Positive (n = 54)	Negative (n = 176)	
Plasma AFP (ng/mL)	465.22 ± 497.11	289.84 ± 252.90	**0.001**	313.96 ± 331.29	302.37 ± 270.47	0.674
Plasma Gal-3BP (µg/mL)	9.23 ± 4.85	8.67 ± 4.80	0.640	8.31 ± 4.32	8.85 ± 4.94	0.322
Plasma HSP70 (ng/mL)	44.66 ± 27.63	42.27 ± 30.98	0.495	40.79 ± 29.84	42.99 ± 30.96	0.736
Plasma kallistatin (µg/mL)	12.57 ± 2.89	16.74 ± 4.08	** < 0.001**	14.16 ± 3.82	17.06 ± 4.02	** < 0.001**
Plasma lipocalin-2 (ng/mL)	131.82 ± 134.13	105.46 ± 93.69	0.138	111.41 ± 113.65	106.63 ± 92.69	0.693
Plasma PSG1 (µg/mL)	7.46 ± 6.35	8.25 ± 5.43	0.286	7.12 ± 5.36	8.50 ± 5.52	0.062
Plasma S100A8 (ng/mL)	785.39 ± 308.84	739.33 ± 312.67	0.654	767.56 ± 314.51	735.88 ± 311.67	0.537
Plasma TGFBI (µg/mL)	2.83 ± 0.64	3.15 ± 0.76	0.084	2.82 ± 0.66	3.22 ± 0.76	** < 0.001**

*AFP* Alpha-fetoprotein, *Gal-3BP* Galectin-3 binding protein, *HSP* Heat shock protein, *PSG1* Pregnancy-specific beta-1 glycoprotein 1, *S100A8* S100 calcium-binding protein A8, *TGFBI* Transforming growth factor beta-induced. Data are given as mean ± standard deviation.

Significant values are in bold.

**Table 3:** Relationship of various plasma proteins with the presence of microbial invasion of the amniotic cavity (MIAC) and intra-amniotic inflammation (IAI), analyzed using multiple logistic regression.

Variables	MIAC^a^		IAI^b^	
	OR (95% CI)	*P-*value	OR (95% CI)	*P-*value
Plasma AFP (µg/mL)	5.303 (1.775–15.841)	**0.003**		
Plasma kallistatin (µg/mL)	0.770 (0.636–0.932)	**0.007**	0.823 (0.751–0.901)	** < 0.001**
Plasma PSG1 (µg/mL)			0.954 (0.895–1.015)	0.138
Plasma TGFBI (µg/mL)	0.443 (0.204–0.961)	**0.039**	0.454 (0.281–0.731)	**0.001**
Serum CRP (mg/dL)	1.729 (1.344–2.224)	** < 0.001**	1.666 (1.314–2.113)	** < 0.001**

*OR* Odds ratio, *CI* Confidence interval, *AFP* Alpha-fetoprotein, *PSG1* Pregnancy-specific beta-1 glycoprotein 1, *TGFBI* Transforming growth factor beta-induced, *CRP* C-reactive protein. ^a^ Adjustment for gestational age at sampling. ^b^ Adjustment for parity.

Significant values are in bold.

**Figure 2:**
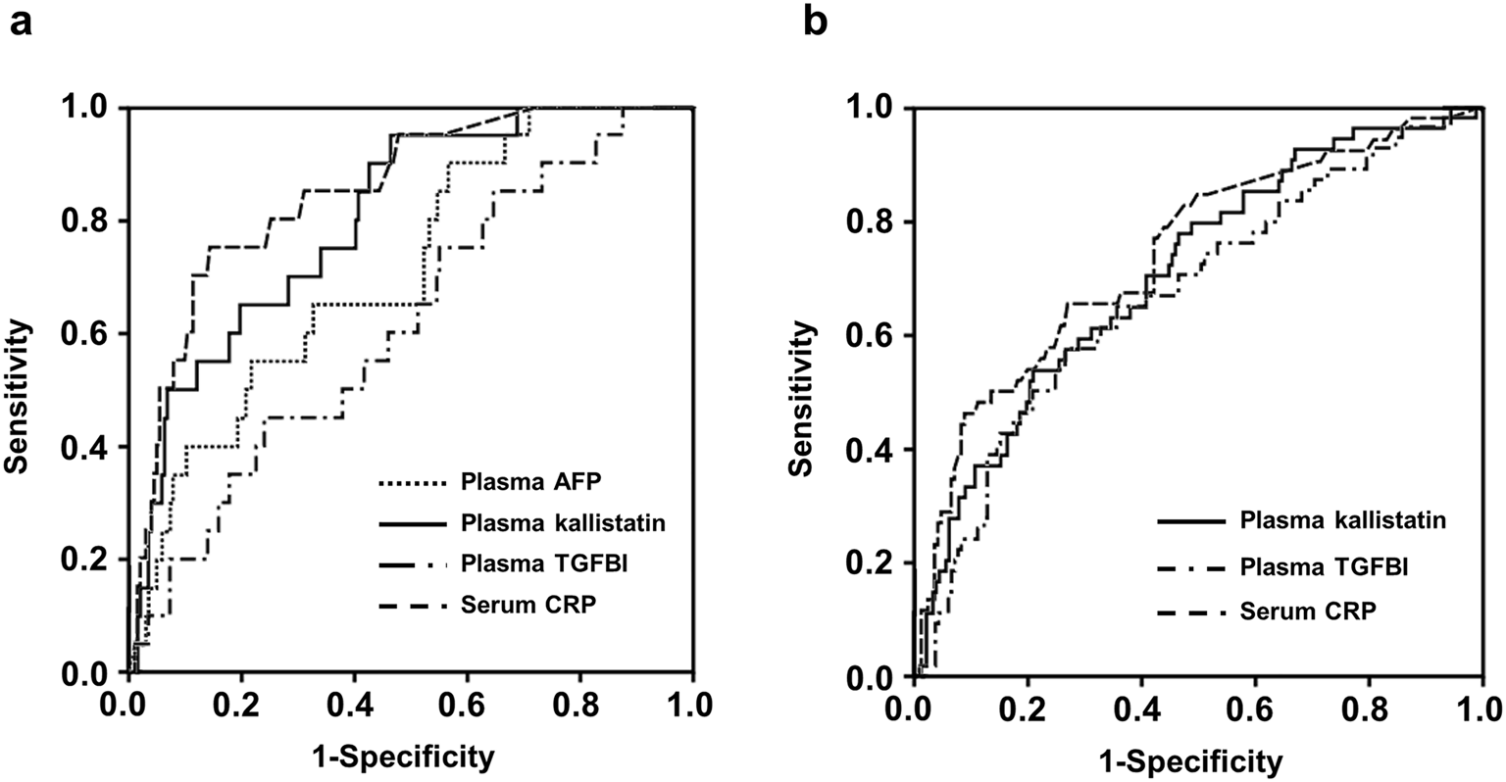
(**a**) Receiver-operating characteristic (ROC) curves of plasma AFP, kallistatin, TGFBI, and serum CRP levels at detecting MIAC (AFP: AUC = 0.72, SE = 0.06, *P* = 0.001; kallistatin: AUC = 0.80, SE = 0.05, *P* < 0.001; TGFBI: AUC = 0.62, SE = 0.06, *P* = 0.084; and CRP: AUC = 0.85, SE = 0.04, *P* < 0.001; (**b**) ROC curves of plasma kallistatin, TGFBI, and serum CRP levels at detecting IAI (kallistatin: AUC = 0.71, SE = 0.04, *P* < 0.001; TGFBI: AUC = 0.67, SE = 0.04, *P* < 0.001; and CRP: AUC = 0.74, SE = 0.04, *P* < 0.001). *AFP* Alpha-fetoprotein, *CRP* C-reactive protein, *MIAC* Microbial invasion of the amniotic cavity, *AUC* Area under the curve, *SE* Standard error, *TGFBI* Transforming growth factor beta-induced, *IAI* Intra-amniotic inflammation.

The areas under the curves (AUCs) values of plasma AFP, kallistatin, and TGFBI for the prediction of MIAC were 0.72, 0.80, and 0.617, respectively (Table 4 and Fig. 2a). The AUCs of plasma kallistatin and TGFBI were not significantly different from those of plasma AFP (*P* = 0.222 and 0.325, respectively) for the prediction of MIAC, whereas the AUC of plasma kallistatin was significantly larger than that of plasma TGFBI (*P* = 0.022). Moreover, the AUC value of plasma kallistatin was not significantly different from that of serum CRP for detecting MIAC (*P* = 0.342), whereas the AUC values of plasma AFP and TGFBI were significantly smaller than those of CRP (*P* = 0.026 and 0.005, respectively).

**Table 4:** Diagnostic indices of various significant plasma biomarkers to predict microbial invasion of the amniotic cavity and intra-amniotic inflammation.

Variables	Area (± SE) under the ROC curve	95% CI	Cut-off value^a^	Sensitivity^b^ (95% CI)	Specificity^b^ (95% CI)	PPV	NPV
Microbial invasion of the amniotic cavity							
Plasma AFP (µg/mL)	0.72 ± 0.06^e^	0.61–0.82	0.29	65.0 (40.8–84.6)	67.6 (60.8–73.9)	16.1	95.3
Plasma kallistatin (µg/mL)	0.80 ± 0.05^e^	0.71–0.89	13.41	65.0 (40.8–84.6)	80.0 (73.9–85.2)	23.6	96.0
Plasma TGFBI (µg/mL)	0.62 ± 0.06	0.49–0.74	2.96	60.0 (36.1–80.9)	54.3 (47.3–61.2)	11.1	93.4
Serum CRP (mg/dL)	0.85 ± 0.04	0.77–0.93	0.64	80.0 (56.3–94.3)	74.9 (68.3–80.7)	23.9	97.4
Combined model A^c^	0.86 ± 0.04	0.79–0.94	0.07	80.0 (56.3–94.3)	74.9 (68.3–80.7)	23.9	97.4
Intra-amniotic inflammation							
Plasma kallistatin (µg/mL)	0.71 ± 0.04f.	0.63–0.79	13.87	53.7 (39.6–67.4)	79.6 (72.8–85.2)	44.6	84.9
Plasma TGFBI (µg/mL)	0.67 ± 0.04f.	0.58–0.75	2.76	57.4 (43.2–70.8)	73.3 (66.1–79.7)	39.7	84.9
Serum CRP (mg/dL)	0.74 ± 0.04	0.66–0.82	0.53	65.4 (50.9–78.0)	73.1 (65.8–79.6)	42.5	87.4
Combined model B ^d^	0.79 ± 0.04	0.72–0.86	0.20	75.0 (61.1–85.9)	68.4 (60.9–75.3)	41.9	90.0

*SE* Standard error, *ROC* Receiver-operating characteristic, *CI* Confidence interval, *PPV* Positive predictive value, *NPV* Negative predictive value, *AFP* Alpha-fetoprotein, *TGFBI* transforming growth factor beta-induced, *CRP* C-reactive protein. ^a^ Cut-off values corresponding to the highest sum of sensitivity and specificity or the point closest to (0, 1) on the ROC curve. ^b^ Values are given as % (95% CI). ^c^ Combined model A consists of plasma kallistatin and serum CRP levels. ^d^ Combined model B consists of plasma kallistatin and TGFBI and serum CRP levels. ^e^* P* < 0.05 compared with Combined model A by the method of DeLong et al. ^f^* P* < 0.005 compared with Combined model B by the method of DeLong et al.

### Verification of proteomic data in relation to IAI in the total cohort

Median plasma levels of kallistatin and TGFBI were significantly lower in women with IAI than in those without this condition (Table 2). Moreover, plasma PSG1 tended to be lower in women with IAI, but this difference was not statistically significant (*P* = 0.062). Additional multivariate analyses revealed that low plasma levels of kallistatin and TGFBI (but not PSG1) were significantly associated with IAI after adjusting for parity (Table 3). However, no association between AFP, Gal-3BP, HSP70, lipocalin-2, and S100A8 and IAI was observed in the plasma of women with PTL. For the detection of IAI, the AUC values of plasma kallistatin and TGFBI were 0.71 and 0.67, respectively (Table 4 and Fig. 2b), and did not differed significantly between them (*P* = 0.488) or when compared with those of serum CRP (*P* = 0.454 for kallistatin and *P* = 0.248 for TGFBI).

Additional univariate analyses of the plasma biomarkers for each outcome measure upon exclusion of data from the patients enrolled in the discovery study (*n* = 210) provided results similar to those observed in the total cohort (*n* = 230), with the exception of AFP that was borderline significantly associated with MIAC (*P* = 0.080) (Tables S4 and S5). No correlations were observed among the measured plasma proteins that were significantly associated with MIAC/IAI (AFP, kallistatin, TGFBI, and CRP) (all variables, *r* =  − 0.079 to 0.012, *P* > 0.2), except for the levels of kallistatin in the plasma, which were significantly correlated with TGFBI levels (*r* = 0.145, *P* = 0.028) or CRP (*r* =  − 0.330, *P* < 0.001).

### Development of multiple-biomarker panels

Overall, plasma kallistatin and CRP levels were identified as the best combination for the identification of MIAC (Table S6), with an AUC value of 0.86 (95% confidence interval [CI]: 0.79–0.94; *P* = 0.933 by Hosmer–Lemeshow test), which was significantly greater than that of plasma kallistatin alone (*P* = 0.022), but not significantly higher than that of serum CRP alone (*P* = 0.700) (Table 4) (Fig. S2a). A predicted probability threshold of ≥ 0.07 was selected as the optimal cut-off value with 80% sensitivity and 75% specificity to identify the presence of MIAC. Using this threshold value, 30% of patients were classified as having high risk and 70.0% as having low risk for MIAC. Indeed, among the patients who were classified as high risk, the observed MIAC rate was 23.9% *vs.* 2.5% in the low-risk group. The negative predictive value (NPV) was 97.4%, suggesting that this model was effective at identifying women at low risk of MIAC.

Similarly, plasma kallistatin and TGFBI levels along with CRP levels were identified as the best combination for predicting IAI (Table S6), with an AUC value of 0.79 (95% CI: 0.72–0.86; *P* = 0.859 by Hosmer–Lemeshow test). The AUC value of this three-biomarker panel was significantly greater than those of kallistatin and TGFBI alone (*P* = 0.003 and *P* = 0.002, respectively) but not significantly higher than that of CRP (*P* = 0.212) (Table 4) (Fig. S1b). A risk probability of ≥ 0.20 was identified as the optimal cut-off value to stratify the IAI risk, with 75% sensitivity, 68% specificity, a positive predictive value of 41%, and an NPV of 90%. Using this threshold value, 41% and 59% of the pregnant women were classified as having high and low risk for IAI, respectively. Given its relatively high NPV (90%), this model may be useful for the exclusion of patients with IAI.

### Additional assessment of the selected plasma proteins in relation to SPTB

Eight selected plasma proteins were also evaluated regarding their predictive potential for SPTB within 7 days because most patients in the case group (9/10) experienced this complication, whereas all patients in the control group had term deliveries. Of the 230 patients with PTL included in the verification cohort, three were excluded from the analysis of SPTB observed within 7 days due to loss to follow-up (*n* = 3). Within 7 days of sampling, SPTB occurred in 56 women (24.6%, 56/227).

Univariate, multivariate, and receiver operating characteristic (ROC) curve analyses confirmed that the results for the prediction of SPTB within 7 days were similar to those for MIAC or IAI (Tables S7, S8, and S9). High levels of AFP and low levels of kallistatin and TGFBI in the plasma were independently associated with the occurrence of SPTB within 7 days in women with PTL (Table S8), with AUCs of 0.58, 0.80, and 0.64, respectively (Table S9 and Fig. S3).

## Discussion

In the present study in women with PTL we have found (i) 133 relevant plasma DEPs and their potential biological pathways that were associated with concomitant MIAC/IAI (or SPTB) and (ii) further validated these proteomic findings for 8 DEPs by immunoassays. In particular, AFP was found to be elevated, whereas kallistatin and TGFBI levels were decreased in the plasma of women with MIAC, and the levels of kallistatin and TGFBI were found to be decreased in the plasma of women with IAI compared with women without these conditions. Furthermore, we found elevated levels of AFP and decreased levels of kallistatin and TGFBI in the plasma of women with SPTB within 7 days of sampling, regardless of their baseline clinical characteristics. However, despite these significant associations, the herein identified plasma biomarkers alone exhibited fair-to-moderate predictive performances for PTL-related MIAC/IAI, as well as SPTB within 7 days (range, 0.58–0.80), which were similar or worse than that of serum CRP (a prototype of inflammatory marker). Our data provide useful insights into the potential molecular mechanisms and biomarkers by which infectious/inflammatory processes in the amniotic cavity and SPTB events are biochemically reflected in circulation.

Over the past decade, several studies have employed proteomic techniques to identify biomarkers in AF and vaginal/cervical fluid samples from women with PTL for the detection of MIAC and/or IAI. Indeed, they have identified several proteins in the amniotic and cervicovaginal fluids that are differentially expressed between MIAC/IAI and non-MIAC/IAI patients, including calgranulins, haptoglobin, insulin-like growth factor-binding protein (IGFBP)-1, lipocalin-2, α1-acid glycoprotein, fatty acid binding protein, and myeloperoxidase^31–36^. However, to the best of our knowledge, no study has used proteomic techniques on plasma or serum samples to identify potential biomarkers of MIAC or IAI in the context of PTL. Using plasma proteomics followed by immunoassays for validation, we discovered, for the first time, three potential new plasma biomarkers (AFP, kallistatin, and TGFBI) that are associated with MIAC/IAI (as well as SPTB) complicated by PTL.

AFP, the fetal form of serum albumin in adults, is a glycoprotein primarily produced by the fetal liver and yolk sac, which crosses the placenta and enters the maternal circulation^37,38^; consequently, the majority of AFP in the maternal blood is of fetal origin^38^. Moreover, maternal serum concentrations of AFP are increased in conditions associated with placental damage and ischemia (such as chorioamnionitis, inadequate angiogenesis [preeclampsia], and fetal growth restriction), which lead to an increased transfer of AFP from the fetus to maternal circulation^39,40^. In the context of asymptomatic first- and second-trimester pregnancies (at 11–13, 15–20, and 24–28 weeks), previous reports have shown that serum AFP concentrations are significantly elevated in women who subsequently develop spontaneous and medically indicated PTB compared with those who deliver at term^41–43^. In the context of experimentally induced ascending placentitis, animal experiments using horses demonstrated that AFP concentrations are significantly elevated in the blood of the ascending placentitis group compared with controls^44,45^. However, to our knowledge, no data in humans are currently available concerning altered AFP expression in the maternal plasma associated with MIAC, IAI, or SPTB in the PTL setting. Overall, consistent with the aforementioned previous studies, the present study demonstrated that plasma AFP levels are significantly elevated in women with PTL who spontaneously delivered within seven days and in those with MIAC.

Kallistatin (also called SERPINA4), which was first discovered in human plasma as a tissue kallikrein-binding protein, is a specific inhibitor of tissue kallikrein that binds to its active site domain^46^. This protein plays a pleiotropic role in human immunopathology, independent of the interaction between kallistatin and tissue kallikrein, such as vasodilation, and inhibition of inflammation, apoptosis, oxidative stress, and angiogenesis^46,47^. In particular, kallistatin acts as a negative acute phase protein that rapidly reduces its expression after lipopolysaccharide-induced inflammation in the liver, contributing to the decreased levels of kallistatin observed in the serum/plasma of patients with inflammation-related conditions, such as obesity, inflammatory bowel disease, and septic syndrome^46,48,49^. In line with inflammatory disease-related data^46,48,49^, our previous studies on pregnancies complicated by PTL or PPROM also showed that kallistatin levels are significantly decreased in the plasma of women with acute HCA or microbial-associated HCA^50,51^. Overall, the findings from the aforementioned studies^63,64^, as well as the inflammatory biological properties and expression profiles of kallistatin, are consistent with and support the herein described association between plasma kallistatin levels and MIAC, IAI, or SPTB risk outcome, given the reported relationships between these endpoints and acute HCA^4,52,53^. Taken together, our present and previous studies suggest that kallistatin may be an important novel protein that efficiently reflects both inflammation/infection in utero and SPTB in the blood compartment.

TGFBI, also known as βig-h3, is a 68-kDa extracellular matrix protein induced by TGF-β in several cell types, including peripheral blood mononuclear cells, keratinocytes, and fibroblasts^54,55^. The precise biological function of TGFBI remains unclear, although it serves as a versatile protein with wide-ranging effects on physiological and pathological conditions, including inflammation, angiogenesis, tumorigenesis, inhibition of cell adhesion, and corneal dystrophy^55^. Importantly, in uterine overdistension conditions (such as polyhydramnios and twins), *TGFBI* mRNA is significantly overexpressed in human uterine myometrium specimens of women with early preterm labor compared with those not in labor^56^, suggesting a significant role of TGFBI in the regulation of preterm parturition. Similarly, in our previous study of PTL, we found that TGFBI levels are significantly elevated in the AF of patients with MIAC, IAI, or SPTB^57^. However, to date, no data is available on the altered expression of TGFBI in plasma or serum samples related to MIAC, IAI, or SPTB in the context of PTL. The present study demonstrated, for the first time, that TGFBI levels are significantly decreased in the plasma of patients with PTL complicated by MIAC, IAI, or SPTB. Nevertheless, this finding contrasts with the data of the aforementioned report on AF samples^57^, although this may be attributed to a scenario (plasma *vs*. AF) reflecting the differences between systemic and local inflammatory responses following in utero infection/inflammation in PTL. Although we did not find data on this issue in the literature, the following evidence may support our finding on plasma TGFBI: 1) patients with type 1 diabetes, of which pathogenesis is involved in a low grade inflammatory process, have significantly decreased levels of TGFBI, but elevated levels of CRP and serum amyloid protein A^58^; 2) serum levels of TGF-β, as an inducible protein for TGFBI, were also significantly lower in women in the preterm birth group compared with controls^70^; and 3) TGFBI was reported to be an important negative regulator of TLR-induced inflammation^71^. Further studies are warranted to explore the underlying mechanism by which the downregulation of TGFBI expression in circulation is associated to MIAC, IAI, or SPTB in women with PTL.

In the present study, IPA allowed the identification of the most important pathways activated in the maternal plasma that are potentially involved in MIAC/IAI and SPTB, including APR signaling, LXR/RXR activation, FXR/RXR activation, IL-12 signaling and production in macrophages, and primary immunodeficiency signaling. These biological pathways are generally consistent with those associated with acute HCA and MIAC/IAI, as reported in previous high-throughput studies using pooled plasma samples collected from women with PPROM^30,59^. The APR signaling pathway is associated with the immune inflammatory response triggered by infection, tissue injury, or trauma, and is mediated by acute phase proteins, which are released from the liver into the blood in response to pro-inflammatory cytokines^60^. RXR functions as a heterodimer with other nuclear receptors, such as LXR and FXR, which is called “LXR/RXR activation” and “FXR/RXR activation”, respectively^61,62^. LXR/RXR activation is involved in the regulation of immune and inflammatory responses in macrophages, as well as lipid, glucose, and cholesterol metabolism^61^. Similar to LXR/RXR activation, FXR/RXR activation is also linked to signaling pathways involved in bile acid, lipid, and glucose metabolism, as well as in the regulation of the inflammatory response^62^. In fact, previous studies using maternal blood or AF samples showed that patients with SPTB and intra-amniotic infection/inflammation have decreased glucose levels and dyslipidemia (i.e., abnormal metabolism)^63–67^. IL-12, a heterodimeric pro-inflammatory cytokine produced by antigen-presenting cells (particularly macrophages) is involved in the host defense against intracellular microbial infections via innate and adaptive mechanisms^68^. Primary immunodeficiency signaling is associated with defects in the expression of molecules related to the development and/or function of the immune system (i.e., defects in innate and adaptive immunity), which are expressed in frequent and severe infections, allergies, and autoimmune disorders^69^. Taken together, the present data on signaling pathways using IPA suggest that MIAC, IAI, and SPTB complicated by PTL are most importantly associated with altered immune regulation and metabolic function that occur in the maternal blood compartment.

The strengths of the study include the following: (i) a relatively large sample size; (ii) this is the first comprehensive study to characterize maternal plasma proteome profile associated with MIAC/IAI in women with PTL using a high-throughput shotgun approach; and (iii) the discovery of protein biomarkers in the plasma specific to MIAC/IAI, despite proteome analysis in blood can be very challenging due to the wide dynamic range of proteins, presence of high-abundance proteins, and biological variability in such biological samples^70^. Our study had several limitations. First, the diagnosis of MIAC was based on the conventional culture identification method, but did not include polymerase chain reaction targeting *16S* rRNA sequence since this diagnostic tool was not covered by public health insurance in Korea. Thus, several microbial infections remain undetected, resulting in an underestimation of the true MIAC rate^6^. Second, this study used long-term stored (− 70 °C) plasma samples, which may have affected the proteomic profiles owing to proteome degradation during the long periods of storage^71^. Third, during the verification phase, the associations of three (37%) of the eight proteins herein identified by global proteome analysis for concomitant MIAC/IAI were replicated by quantitative ELISA using individual plasma samples of the total cohort. Particularly, while the shotgun proteomic analyses revealed upregulated expression of kallistatin and TGFBI in the pooled plasma samples of case group, this was reversed in the individual samples analyzed by ELISA. Fourth, our discovery proteomic analysis was performed using pooled plasma samples for each group, which have the following inherent shortcomings: (i) lack of representation of the biological average of the individual samples, and (ii) missed and/or false identification of makers (than when using individual samples) because of the reduced statistical value of the identified markers^72^. Fifth, the spectral counting-based label-free quantification method used herein was inherently biased against low-abundance proteins during MS/MS data acquisition, thereby decreasing the overall accuracy and sensitivity of the quantification, which may interfere with the detection of biologically important proteins^73^. Sixth, the prevalence of MIAC, IAI, and SPTB within 7 days of sampling was relatively low in the present study (8.6% [20/230], 23.4% [54/230], and [24.6%, 56/227], respectively) as compared with that previously reported in the published literature (10–20% for MIAC, 30–50% for IAI, and 10–30% for SPTB)^2–6,74–77^. Of note, the prevalence of these pregnancy complications may be affected by several factors including detection methods (conventional culture vs. molecular methods), definition of IAI, GA at which the sampling was performed, and the threshold required to perform amniocentesis in women with PTL at different hospitals. Seventh, a uniform treatment was not applied to all women with PTL since (i) the study cohort included patients treated in a single hospital over a 16-year time period and (ii) several attending obstetricians cared for patients during this period. These factors may have influenced to different degrees the various plasma proteins and the outcomes of interest. Finally, this study had a retrospective design, and the validations of the eight selected candidate proteins and our combined prediction models were not performed in a completely independent set of samples. All these points may limit the generalizability of our findings, which may warrant further validation in other cohort.

## Conclusions

In conclusion, the proteomic analysis of plasma from women with PTL led to the identification of 133 DEPs and their biological pathways associated with concomitant MIAC/IAI (or SPTB). We validated our proteomic data using ELISA and identified some proteins (i.e., AFP, kallistatin, and TGFBI) present in the maternal plasma that were independently associated with MIAC, IAI, or SPTB. In the context of PTL, the development of non-invasive tests for identifying patients at high risk of MIAC, IAI or SPTB would improve pregnancy outcomes through increased clinical surveillance and the targeted use of novel therapeutics (particularly clarithromycin). In light of these issues, further studies are required to (i) examine whether these biomarkers assessed in other non-invasive samples, such as vaginal fluid, saliva, or urine, could also hold potential for identifying patients at high risk of developing MIAC, IAI, or SPTB; and (ii) evaluate whether combining these plasma biomarkers with vaginal fluid-based tests and clinical parameters (e.g*.*, cervical length, cervicovaginal fluid IGFBP-1, AFP, C3a, C5a, and IL-6 levels) could significantly improve the predictive potential for MIAC, IAI, or SPTB, as a recently reported by Cobo *et al*^24,78–80^.

## Materials and methods

### Ethical approval

This study was approved by the ethics committee of Seoul National University Bundang Hospital, Seongnamsi, Korea (project number B-1105/128-102). All experiments were performed in accordance with the relevant guidelines and regulations of the hospital ethics committee. Written informed consent was obtained from all participants for the collection and use of biological samples before amniocentesis.

### Study population and design

This retrospective cohort study involved 230 singleton pregnant women who were admitted to Seoul National University Bundang Hospital (Seongnamsi, Korea) between June 2004 and February 2021 and were diagnosed with PTL and intact membranes at 24 + 0 to 34 + 0 weeks of gestation. Participants were selected from a comprehensive perinatal database of high-risk pregnancies that was prospectively compiled and maintained since 2004. Eligible patients were identified according to the following inclusion criteria: (i) performance of amniocentesis to evaluate subclinical infection/inflammation in the amniotic cavity, (ii) availability of an aliquot of plasma samples collected at the time of amniocentesis, (iii) absence of active labor on recruitment (defined as cervical dilation of 4 cm or more), and (iv) delivery of a live fetus. Women with ruptured membranes, multiple pregnancies, clinical signs of chorioamnionitis at presentation, and with fetuses with major congenital anomalies were excluded from the study. The primary endpoint of the study was MIAC or IAI and the secondary endpoint was SPTB within 7 days of sampling. PTL was diagnosed in women requiring hospital admission due to regular and painful uterine contractions occurring at least twice every 10 min, associated with cervical changes (softening, dilation, or effacement) before 37 weeks of gestation. A subset of women with PTL (*n* = 84) was included in a previous study, which identified plasma coagulation factor V and S100A9 as potential novel biomarkers of SPTB with non-IAI etiology^81^.

**Figure 3:**
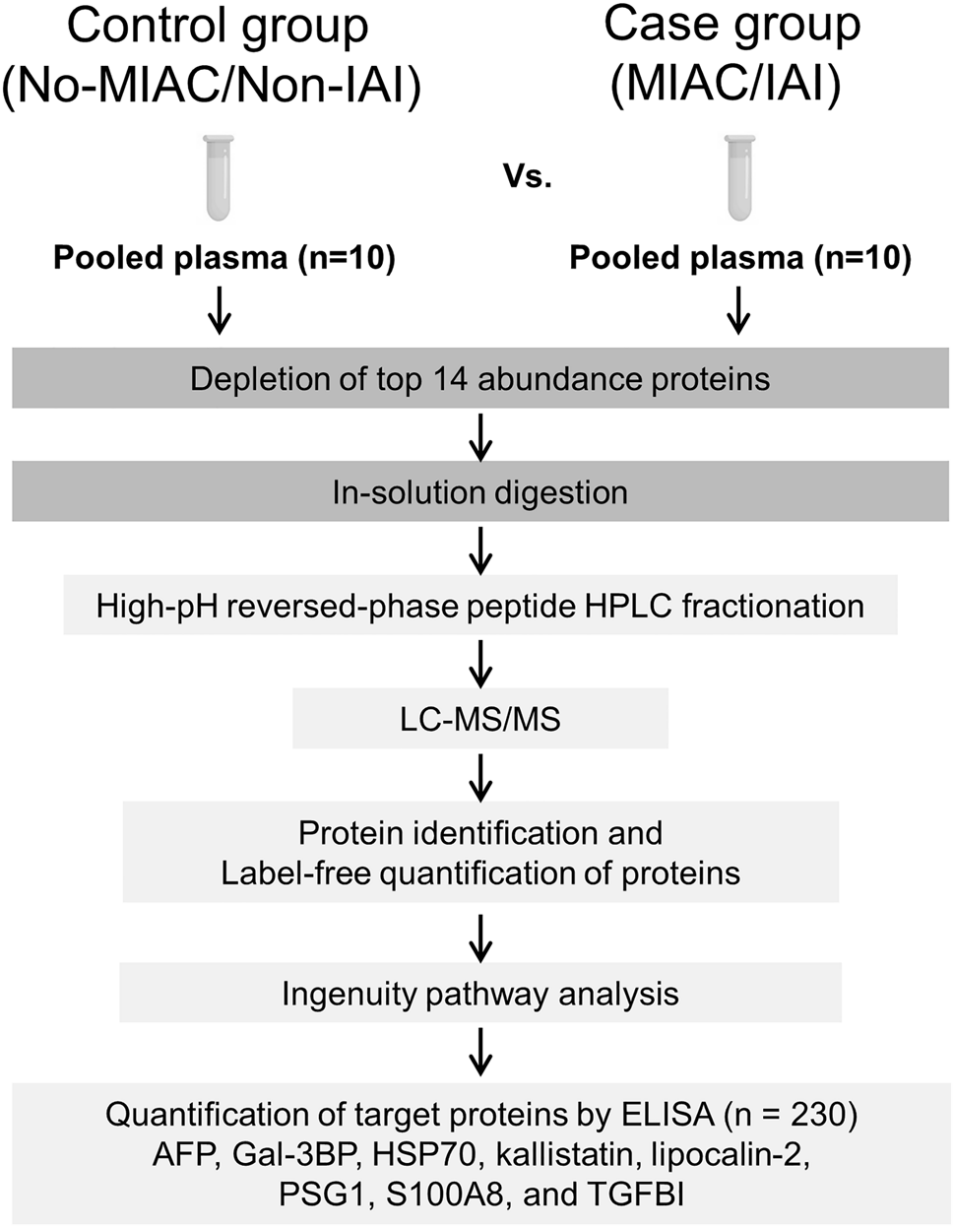
Schematic workflow of the experimental design for proteomics discovery and verification analysis of plasma samples of women with MIAC/IAI in the context of PTL. Plasma samples pooled from case (concomitant MIAC/IAI) and control (non-MIAC/IAI) groups (10 samples per group) were subjected to immunoaffinity depletion to remove 14 high-abundance proteins, followed by tryptic digestion and spectral count-based label-free quantitation method. The identified DEPs were further characterized by IPA and selected DEPs of interest were further verified by ELISA. *MIAC* Microbial invasion of the amniotic cavity, *IAI* Intra-amniotic inflammation, *HPLC* High performance liquid chromatography, *LC* Liquid chromatography, *MS/MS* Tandem mass spectrometry, *IPA* Ingenuity pathway analysis, *DEP* Differentially expressed proteins, *ELISA* Enzyme-linked immunosorbent assay, *AFP* Alpha-fetoprotein, *Gal-3BP* Galectin-3 binding protein, *HSP* Heat shock protein, *PSG1* Pregnancy-specific beta-1 glycoprotein 1, S100A8, S100 calcium-binding protein A8; TGFBI, transforming growth factor beta-induced.

For the exploratory phase using label-free quantitative proteomics (Fig. 3), a nested case–control study comprising 10 patients with both MIAC and IAI (case group: delivered preterm within 7 days of sampling) and 10 GA-matched patients without MIAC and IAI (control group: delivered at term) was performed. The patients in the case group were randomly selected (using a random sequence generator) from a group of 19 patients with both MIAC and IAI within the total study cohort of 230 patients with PTL. Each patient in the non-MIAC/IAI control group was selected for each MIAC/IAI case considering GA at sampling, parity, and duration of plasma sample storage.

### Diagnosis of MIAC and IAI

The amniocentesis and microorganisms detection procedures used, as well as the white blood cell (WBC) counts in the AF and AF IL-6 levels have been previously reported in detail^82,83^. Briefly, transabdominal amniocentesis at the time of admission was performed under ultrasonographic guidance using an aseptic technique. Immediately after amniocentesis, AF samples were cultured for genital mycoplasma (*Mycoplasma hominis* and *Ureaplasma urealyticum*), aerobic and anaerobic bacteria, and fungi, and WBC counts were assessed. The remaining AF samples were centrifuged at 1,500 × *g* at 4 °C for 10 min and the supernatant was aliquoted and stored at − 70 °C until assayed. The managing physicians had access to the AF microbial culture and WBC count results.

IL-6 levels were measured by ELISA using Human IL-6 DuoSet Kits (R&D Systems, Minneapolis, MN, USA) in previously stored AF samples (a detailed description is provided in Supplementary Materials). The minimum detectable IL-6 concentration was 9.4 pg/mL. IAI was defined as the presence of high levels of AF IL-6 (> 2.6 ng/mL) as previously described^4,84^. Information on IL-6 levels in AF was not available to the managing clinicians. MIAC diagnosis was confirmed based on positive results for bacteria, fungi, and/or genital mycoplasmas in the AF, as detected using the conventional culture method.

### Collection and storage of plasma samples

On admission, the patients underwent amniocentesis and venous blood samples were collected to perform routine blood tests, including analysis of CRP levels and WBC counts, as per the hospital protocol. Additional blood samples were collected in tubes containing ethylenediaminetetraacetic acid solution, which were then centrifuged at 1500 × g at 4 °C for 10 min and then stored in multiple aliquots at − 70 °C until use. Significantly hemolyzed plasma samples were excluded from the study. Blood samples were, in general, collected before administering any medications (antibiotics, corticosteroids, or tocolytics).

### Clinical management of PTL and definition of various factors

The protocols for the management for PTL and for the diagnosis of acute HCA and clinical chorioamnionitis have been previously described in detail (Supplementary Materials)^59,81^. Briefly, corticosteroids and tocolytic therapy (magnesium sulfate, atosiban, or ritodrine) were administered to women with PTL at a GA of 23–34 weeks at the discretion of the attending physician if there was no clinical contraindication. Prophylactic broad-spectrum antibiotics (including ampicillin/amoxicillin plus macrolides) were not administered to any women with PTL to prolong pregnancy, except for the following conditions: clinically suspected or diagnosed subclinical MIAC/IAI and development of clinical signs of chorioamnionitis. However, clinical decisions regarding the antibiotics type and treatment duration, as well as on delivery timing in patients with PTL complicated with or suspected of MIAC/IAI were primarily made at the discretion of the attending obstetricians. In general, women with PTL and culture-proven MIAC at < 34 weeks of gestation were not recommended to deliver solely based on positive AF cultures and were administered antibiotics (which were selected based on the AF culture and antibiotic sensitivity results) until 34 + 0 weeks, after which they delivered.

### Label-free quantitative analysis (discovery phase)

Protein concentrations in each of the 20 discovery cohort samples were determined using a bicinchoninic acid assay (Micro BCA Protein Assay Kit; Thermo Fisher Scientific, Bremen, Germany) (Fig. 3). Equal amounts of protein (400 μg) from each of the MIAC/IAI cases and non-MIAC/IAI controls were pooled separately to produce a single sample from each group (4 mg per group). The pooled plasma samples were then subjected to immunoaffinity depletion, tryptic digestion, and high-pH reversed-phase peptide fractionation, and analyzed in triplicate using an online Thermo Easy nLC 1000 system interfaced with a Thermo quadrupole-orbitrap Q-Exactive mass spectrometer controlled by Xcalibur version 2.0.6 software (all from Thermo Fisher Scientific, Waltham, MA, USA) (see Supplementary Materials).

For peptide identification, raw mass spectrometry files were searched against the UniProt protein database (42,083 entries; released March 2015) using the SEQUEST search algorithm (Sorcerer v 4.3.0; Sage-N Research, Milpitas, CA, USA). The SEQUEST search parameters were as follows: fully tryptic peptides, missed cleavages of two, parent mass tolerance of 10 ppm, fragment mass tolerance of 1 Da, fixed modifications of carbamidomethyl cysteine (+ 57.021 Da), and variable modifications of methionine oxidation (+ 15.995 Da). Scaffold 4 (version 4.3.2; Proteome Software Inc., Portland, OR, USA) was used to filter tandem mass spectrometry (MS/MS)-based peptide and protein identification. Protein and peptide identification was accepted if it could be established at < 1% false discovery rate and the protein contained at least two unique peptides. Only proteins identified in at least two technical replicates were included in the statistical analyses. The spectral counts from the MIAC/IAI cases and non-MIAC/IAI controls were log_2_-transformed and compared using the R statistical software with a PLGEM (http://www.bioconductor.org) to identify DEPs between the control and case groups^85–87^. The relative abundance ratios of proteins that exhibited statistically significant changes were calculated as the ratios between spectral counts in MIAC/IAI cases and those in non-MIAC/IAI controls. Proteins with FCs > 1.5 or < 0.66, either up- or downregulated, with a *P*-value < 0.05, were considered DEPs. Only the proteins that met these criteria were subjected to further analysis.

### Ingenuity pathway analysis (*IPA*)

Web-based IPA software (data version 84,978,992; QIAGEN, Redwood City, CA, USA) (https://digitalinsights.qiagen.com) was used for functional analysis to identify the canonical pathways, diseases, and biological functions in which the identified DEPs were involved. UniProt accession numbers of the DEPs and their corresponding log_2_ ratios between spectral counts from MIAC/IAI and non-MIAC/IAI groups were uploaded into the IPA software. The uploaded DEPs were mapped to the corresponding gene objects in the Ingenuity Pathway Knowledge Base as a reference set^88,89^. The *P*-value of the IPA analysis was calculated using the right-tailed Fisher's exact test, with a cut-off value of < 0.05.

### Immunoassays (validation phase)

The selected candidate DEPs were evaluated in the study cohort (comprising 230 individual samples) by immunoassays to validate the proteomic data. The concentrations of AFP, Gal-3BP, HSP70, kallistatin, lipocalin-2, PSG1, S100A8, and TGFBI were assayed using commercial ELISA kits (DuoSet ELISA; R&D Systems, Minneapolis, MN, USA). All assays were performed in strict accordance with the manufacturers’ instructions. The working range of each ELISA kit and the corresponding dilution ratios are described in detail in Supplementary Materials. The intra- and inter-assay coefficients of variation were < 15% for all the analyzed proteins. The aforementioned molecules were selected for the validation study based on (i) the magnitude of either FC or significant *P*-values; (ii) little or no information on their altered expression in the plasma associated with MIAC/IAI or preterm birth in the context of PTL; (iii) potential to be reflected in maternal blood when inflammation/infection was present in utero, considering their production site (e.g., liver) and biological role^90^; and (iv) the availability of commercial ELISA kits and their good performance in plasma samples, which was verified using spike-and-recovery and linearity-of-dilution experiments.

### Statistical analysis

The clinical and plasma data were compared using the Fisher’s exact test or *χ*^2^-test for categorical data, Student’s *t*-test for normally distributed continuous data, and the Mann–Whitney *U*-test for non-normally distributed continuous data. Subsequently, multivariate logistic regression analysis was performed to estimate independent associations between the plasma levels of each protein and the outcome measure after adjusting for GA at sampling and parity, which were found to be implicated (*P* < 0.1) in the univariate analyses. Medication (antibiotic and corticosteroid) use was not adjusted in the logistic regression model because most medications were administered after sampling, as previously described; thus, the effect of the plasma biomarkers of interest on the outcome was not confounded by the effect of medication administration. Additionally, to develop novel blood-based multiple-biomarker panels for determining the outcomes of interest, multivariate logistic analyses with forward selection were performed using the newly identified plasma biomarkers (AFP, kallistatin, and TGFBI) along with a prototype inflammatory biomarker in the blood (serum CRP), which showed *P* < 0.1 in the univariate analysis. Diagnostic values of each candidate protein were assessed via ROC curve analyses and AUCs. Thereafter, pairwise comparisons of the AUCs between the investigated plasma biomarkers, serum CRP (as a prototype of inflammatory markers and one of the DEPs), and multiple-biomarker panels were performed using the method proposed by DeLong *et al*^91^. The optimal cut-off value was determined using the point closest to (0, 1) on the ROC curve or maximal Youden's index (sum of sensitivity + specificity − 1)^92^. Spearman’s rank correlation test was used to evaluate the correlation between the concentrations of various plasma proteins. All probability values were two-sided and values of *P* < 0.05 were considered significant. Statistical analyses were performed using the SPSS version 25.0 software (IBM Corp., Armonk, NY, USA).

### Supplementary Information

Supplementary Figures.

Supplementary Tables.

Supplementary Information 3.

## Data Availability

All relevant data are within the paper, and the corresponding author (KHP) can make available materials, data and associated protocols if requested.
