# Supplementary material for: Identification and characterization of plasma proteins associated with intra-amniotic inflammation and/or infection in women with preterm labor
Source: Sci Rep. 2024 Jun 25;14:14654. doi: 10.1038/s41598-024-65616-x (PMC11199617; doi:10.1038/s41598-024-65616-x)

Title page

Identification and characterization of plasma proteins associated with intra-amniotic inflammation and/or infection in women with preterm labor

Hee Young Cho[1†], Ji Eun Lee[2,†], Kyo Hoon Park[3,*], Bo Young Choi[3], Min Jung Lee[3], Da Eun Jeong[3], and Sue Shin[4]

[1] Department of Obstetrics and Gynecology, Seoul National University College of Medicine, Seoul National University Hospital, Seoul, Korea
[2] Chemical & Biological integrative Research Center, Biomedical Research Division, Korea Institute of Science and Technology, Seoul 02792, Korea
[3] Department of Obstetrics and Gynecology, Seoul National University College of Medicine, Seoul National University Bundang Hospital, Seongnam, Korea
[4] Department of Laboratory Medicine, Seoul National University College of Medicine, Seoul National University Boramae Hospital, Seoul, Korea

[†] These two authors contributed equally to this work and should therefore be regarded as equivalent authors.

[*] Corresponding author

Address correspondence to:
Kyo Hoon Park, MD, PhD
Department of Obstetrics and Gynecology
Seoul National University Bundang Hospital
82, Gumi-ro 173 Beon-gil, Bundang-gu, Seongnam, 463-707, Korea
Tel: 82-31-787-7252; Fax: 82-31-787-4054; E-mail: pkh0419@snubh.org

Figure S1. Volcano plot constructed from label-free quantification data

Volcano plot shows the plasma upregulated and downregulated differentially expressed proteins (DEPs) in the MIAC/IAI and non-MIAC/IAI groups. Representative protein identifiers in black font indicate DEPs of interest verified by ELISA.

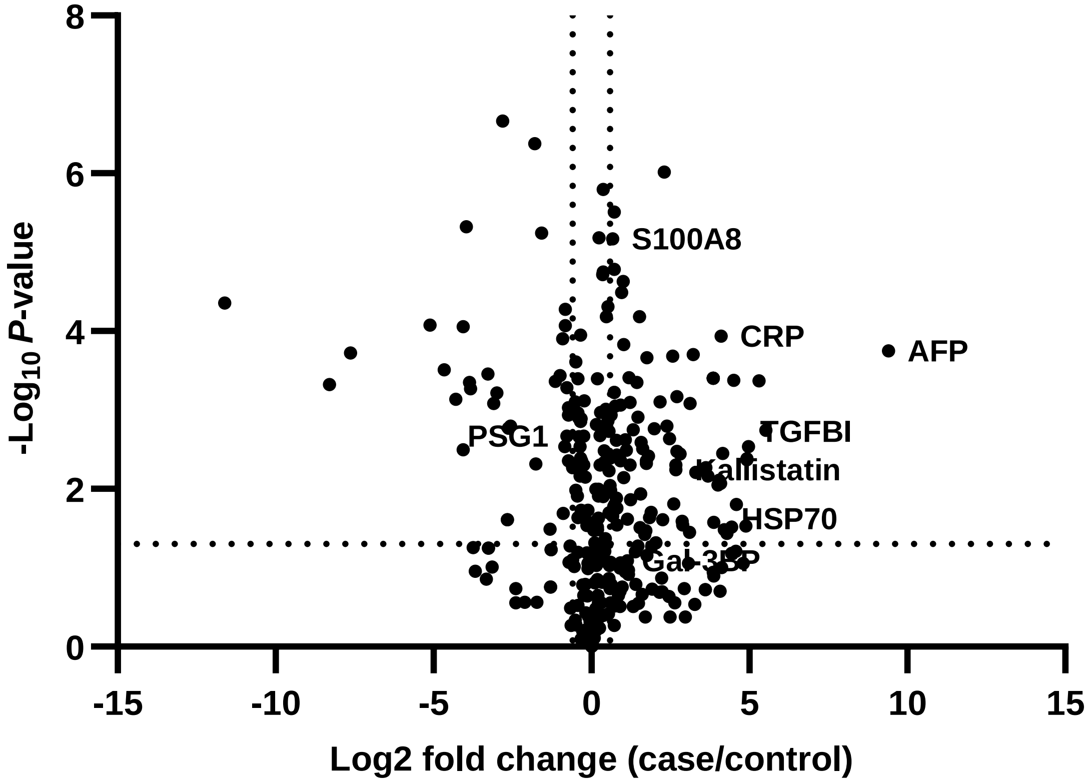

Figure S2. Receiver-operating characteristic (ROC) curves

(a) ROC curve of combination model of plasma kallistatin and serum CRP levels in detecting MIAC. AUC = 0.86, SE = 0.04, P <0.001. (b) ROC curve of combination model of plasma kallistatin and TGFBI and serum CRP levels in detecting IAI. AUC = 0.79, SE = 0.04, P <0.001. CRP, C-reactive protein; MIAC, microbial invasion of the amniotic cavity; AUC, area under the curve; SE, standard error, TGFBI, transforming growth factor beta-induced; IAI, intra-amniotic inflammation.

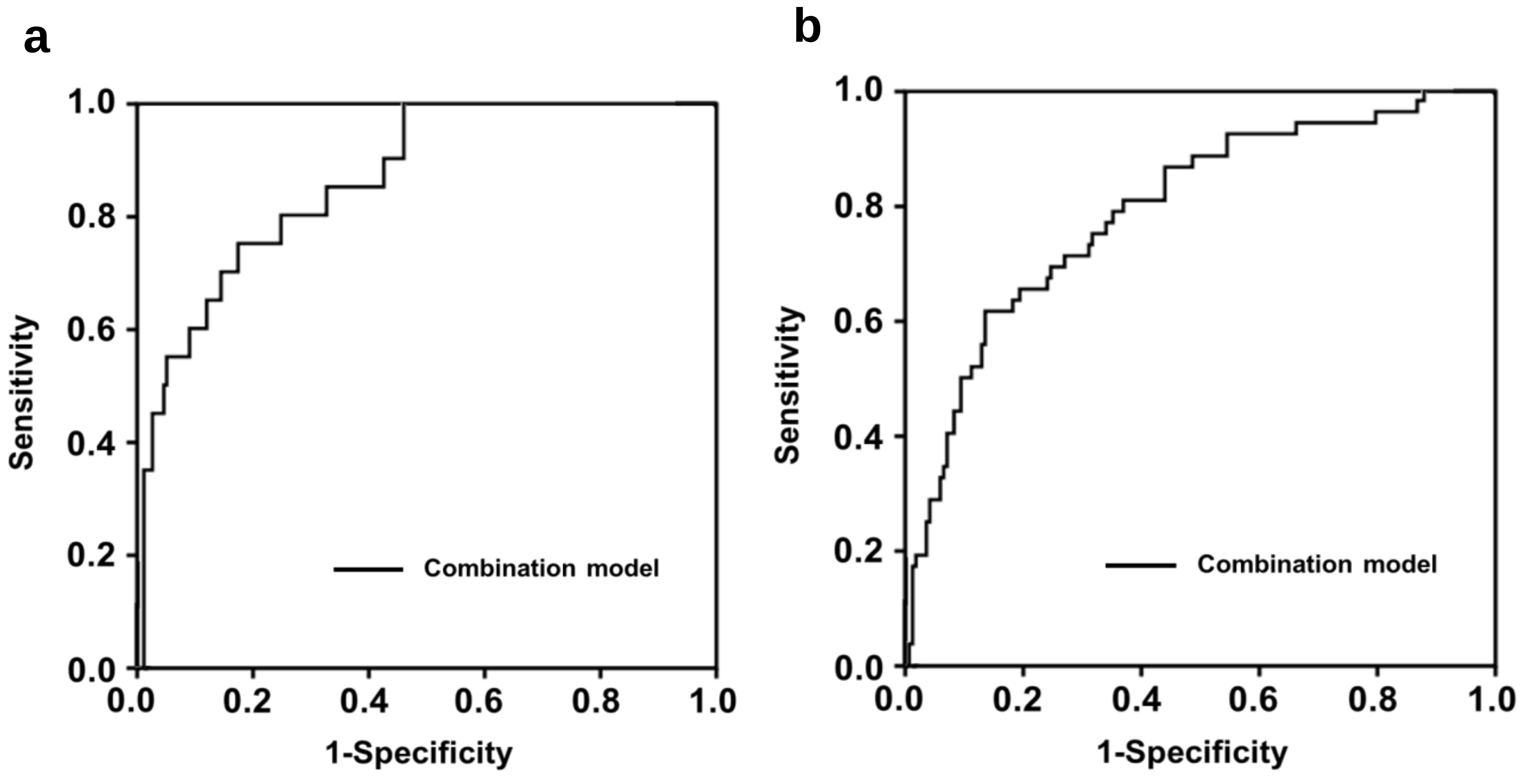

Figure S3. Receiver-operating characteristic (ROC) curves

ROC curves of plasma AFP, kallistatin, TGFBI, and serum CRP levels for predicting SPTB within 7 days of sampling (AFP: AUC = 0.58, SE = 0.05, P = 0.084; kallistatin: AUC = 0.80, SE = 0.03, P < 0.001; TGFBI: AUC = 0.64, SE = 0.05, P = 0.002; and CRP: AUC = 0.72, SE = 0.04, P < 0.001). AFP, alpha-fetoprotein; TGFBI, transforming growth factor beta-induced; CRP, C-reactive protein; SPTB, spontaneous preterm birth; AUC, area under the curve; SE, standard error.

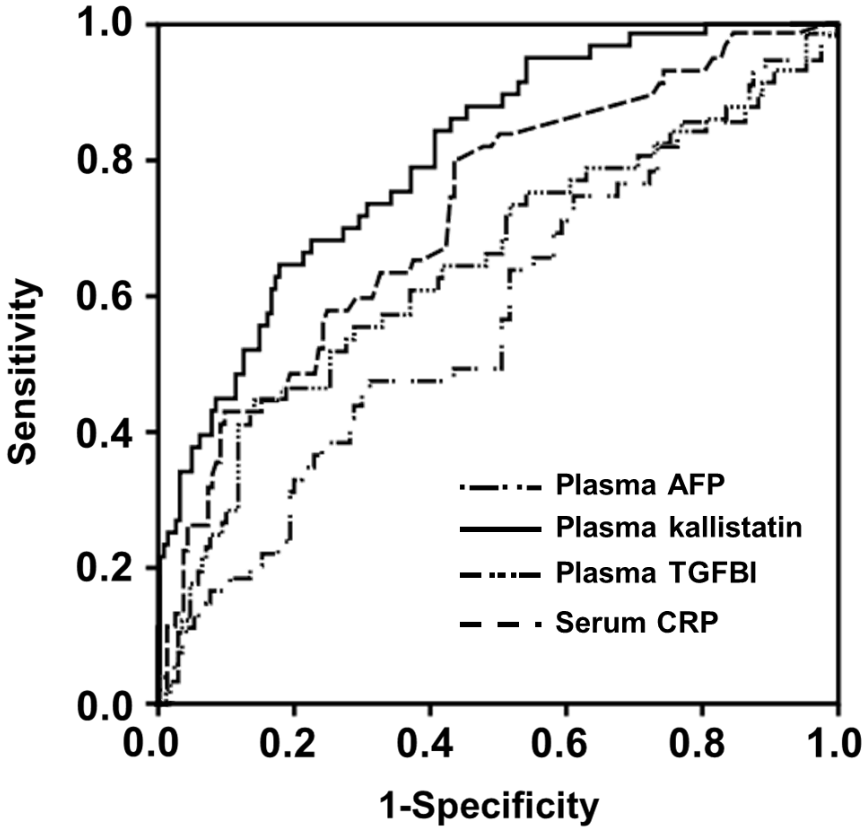

Supplement: Supplementary file 1 — Supplementary Figures. [file 41598_2024_65616_MOESM1_ESM.pdf]
