# Supplementary material for: Identification and characterization of plasma proteins associated with intra-amniotic inflammation and/or infection in women with preterm labor
Source: Sci Rep. 2024 Jun 25;14:14654. doi: 10.1038/s41598-024-65616-x (PMC11199617; doi:10.1038/s41598-024-65616-x)
Supplement: Supplementary file 3 — Supplementary Information 3. [file 41598_2024_65616_MOESM3_ESM.docx]

**Title page**

**Identification and characterization of plasma proteins associated with intra-amniotic inflammation and/or infection in women with preterm labor**

Hee Young Cho^1†^, Ji Eun Lee^2,†^, Kyo Hoon Park^3,^**^*^**, Bo Young Choi^3^, Min Jung Lee^3^, Da Eun Jeong^3^, and Sue Shin^4^

^1^ Department of Obstetrics and Gynecology, Seoul National University College of Medicine, Seoul National University Hospital, Seoul, Korea

^2^ Chemical & Biological integrative Research Center, Biomedical Research Division, Korea Institute of Science and Technology, Seoul 02792, Korea

^3^ Department of Obstetrics and Gynecology, Seoul National University College of Medicine, Seoul National University Bundang Hospital, Seongnam, Korea

^4^ Department of Laboratory Medicine, Seoul National University College of Medicine, Seoul National University Boramae Hospital, Seoul, Korea

^†^ These two authors contributed equally to this work and should therefore be regarded as equivalent authors.

**^*^** Corresponding author

Address correspondence to:

Kyo Hoon Park, MD, PhD

Department of Obstetrics and Gynecology

Seoul National University Bundang Hospital

82, Gumi-ro 173 Beon-gil, Bundang-gu, Seongnam, 463-707, Korea

Tel: 82-31-787-7252; Fax: 82-31-787-4054; E-mail: [pkh0419@snubh.org](mailto:pkh0419@snubh.org)

## - Supplementary Materials –

**Analysis of IL-6 in the AF and diagnosis of acute histologic and clinical chorioamnionitis**

The range of the IL-6 standard curve was 7.8–600 pg/mL. The assay was carried out by strictly following the instructions provided by the manufacturer and samples were measured in duplicate. The calculated intra- and inter-assay coefficients of variation (CV) were <10%. Acute HCA was defined as the presence of neutrophil infiltration involving fetal membranes (amnion and chorion-decidua), chorionic plate, or umbilical cord, according to previously published criteria^1,2^. Clinical chorioamnionitis was diagnosed following the criteria proposed by Gibbs et al.^3^; fever (≥37.8°C) and the presence of two or more of the associated clinical findings (uterine tenderness, malodorous vaginal discharge, maternal leukocytosis, maternal tachycardia, and fetal tachycardia).

**Immunoaffinity depletion of high-abundance proteins in plasma samples**

Top 14 abundant proteins (albumin, alpha-1 acid glycoprotein, alpha-1 antitrypsin, alpha-2 macroglobulin, apolipoprotein A-I, apolipoprotein A-II, complement C3, fibrinogen, haptoglobin, immunoglobulin A, immunoglobulin G, immunoglobulin M, serotransferrin, and transthyretin) were removed from each pooled plasma sample (4 mg per group) using a multiple affinity removal system (MARS) (Hu-14; Agilent Technologies, Santa Clara, CA) according to the manufacturer’s instructions. The depleted plasma samples were dialyzed with 50 mM ammonium bicarbonate buffer (ABC) and then concentrated using Amicon Ultra centrifugal filter units (Ultracel-3K; Millipore, Billerica, MA). Total protein concentration of the depleted plasma samples was determined by BCA assay (Micro BCA Protein Assay Kit, ThermoFisher Scientific, Bremen, Germany).

**In-solution tryptic digestion**

Three hundred micrograms of the protein from the depleted plasma samples in each group was resuspended in 100 μL of 8 M urea buffer and incubated with 3 mM dithiothreitol at room temperature for 1 h for reduction of the disulfide bonds. The samples were then reacted with 5 mM iodoacetamide at room temperature for 1 h in the dark to alkylate the cysteine residues. After the samples were diluted with 50 mM ABC buffer, they were subjected to digestion by trypsin (Promega, Madison, WI, at a ratio of 50 to 1, μg protein to μg trypsin) for 16 h at 37 °C. After trypsin digestion, the peptide solution was acidified with 10% trifluoroacetic acid (TFA), and desalted using a Macro Spin Column (C-18; Harvard Apparatus, Holliston, MA). The peptide samples were dried with a CentriVap® benchtop vacuum concentrator (Labconco, Kansas City, MO), and the peptide concentration was determined using a BCA kit.

**High pH reversed-phase fractionation**

Three hundred micrograms of the peptides obtained from each group were separated by high pH reversed-phase fractionation using Agilent 1260 high-performance liquid chromatography (HPLC) infinity purification system (Agilent Technology, Santa Clara, CA). An Xbridge C-18 column (ZORBAX, 4.6 × 250 mm, 5 μm, 300 Å; Waters, Milford, MA) was equilibrated with 10 mM ammonium formate (AF) in water and serially fractionated with 10 mM AF in acetonitrile (ACN) (15, 28.5, 34, and 60% ACN), with eight elution fractions collected separately. The fractionated peptide samples were dried in Centrivap (Labconco) and the dried peptides were reconstituted with 0.1% formic acid (FA) in water.

**Q Exactive™ hybrid Quadrupole-Orbitrap™ mass spectrometry**

The fractionated peptide samples in each group were analyzed in triplicate using an Easy nano II Ultra Performance Liquid Chromatography 1000 system (Thermo Fisher Scientific, Bremen, Germany) coupled with a Thermo quadrupole-orbitrap Q-Exactive Mass Spectrometer (Thermo Fisher Scientific, Bremen, Germany). Two µL of each peptide fraction was injected into a trap column (C18, 3 μm, 0.7 cm, Thermo Fisher Scientific) and separated on an Acclaim PepMap column (C18, 2 µm, 100 Å, 50 cm, Thermo Fisher Scientific) with a flow rate of 200 nL/min with the following gradient conditions: 0 min 95% buffer A (100% water with 0.1% FA) and 5% buffer B (100% ACN with 0.1% FA), 0−3 min at 5% B, 3−69 min 3−38% B, 69−72 min 38−90% B, 72−82 min at 90% B, 82−85 min 90%−5% B, and 85−100 min at 5% B.

A top 10 method was used for the Q-Exactive mass spectrometer. Full MS spectra (m/z 300 - 1,600 range; resolution, 70,000) were acquired for the peptides using the Orbitrap mass analyzer. The automatic gain control (AGC) target value for the MS data acquisition was 1 X 10^6^. The ten most intense ions were fragmented in the higher-energy collisional dissociation (HCD) collision cell (normalized collision energy of 27%) in data-dependent MS2 acquisition (DDA) mode. The MS/MS spectra were acquired in the Orbitrap mass analyzer (resolution, 17,500; AGC target value, 1 X 10^5^; intensity threshold, 8.3 X 10^3^; maximum allowed ion accumulation time, 50 ms for full MS scans and 120 ms for tandem mass (MS/MS) spectrum). The precursor ions with 1 and 5 or more charge states were excluded for MS/MS scan events. The same precursor ions were also excluded after 20 s using the dynamic exclusion function.

**Analysis of various proteins in the plasma**

The ranges of AFP, Gal-3BP, HSP70, kallistatin, lipocalin-2, PSG 1, S100A8, and TGFBI standard curves were 78.125–5000 pg/mL, 62.5–4000 pg/mL, 125–8000 pg/mL, 125–8000 pg/mL, 78.125–5000 pg/mL, 39–2500 pg/mL, 31.2–2000 pg/mL, and 62.5–4000 pg/mL, respectively. Prior to measurement of these proteins, the maternal plasma samples were diluted at 1:10 for HSP70, 1:100 for lipocalin-2, 1:500 for AFP and S100A8, 1:5000 for TGFBI, and 1:10000 for Gal-3BP, kallistatin, and PSG 1. The intra- and inter-assay coefficients of variation were 5.7% and 11.1% for AFP, 6.8% and 10.2% for Gal-3BP, 1.8% and 8.9% for HSP70, 3.1% and 12.6% for kallistatin, 4.0% and 14.4% for lipocalin-2, 3.0% and 3.8% for PSG 1, 3.3% and 9.3% for S100A8, and 2.2% and 11.7% for TGFBI, respectively.

**Reference**

1. Kim CJ, Romero R, Chaemsaithong P, Chaiyasit N, Yoon BH, Kim YM. Acute chorioamnionitis and funisitis: definition, pathologic features, and clinical significance. Am J Obstet Gynecol. 2015;213:S29-52.
2. Jung EY, Choi BY, Rhee J, Park J, Cho SH, Park KH. Relation between amniotic fluid infection or cytokine levels and hearing screen failure in infants at 32 wk gestation or less. Pediatr Res. 2017;81:349-55.
3. Gibbs RS, Blanco JD, St Clair PJ, Castaneda YS. Quantitative bacteriology of amniotic fluid from women with clinical intraamniotic infection at term. J Infect Dis. 1982;145:1-8.
